# Contextual Barriers to Implementing Open-Source Electronic Health Record Systems for Low- and Lower-Middle-Income Countries: Scoping Review

**DOI:** 10.2196/45242

**Published:** 2024-08-01

**Authors:** Sarah Bostan, Owen A Johnson, Lena J Jaspersen, Rebecca Randell

**Affiliations:** 1 Leeds University Business School University of Leeds Leeds United Kingdom; 2 School of Healthcare University of Leeds Leeds United Kingdom; 3 School of Computing University of Leeds Leeds United Kingdom; 4 Faculty of Health Studies University of Bradford Bradford United Kingdom; 5 Wolfson Centre for Applied Health Research Bradford United Kingdom

**Keywords:** implementation, open source, electronic health records, digital health, low- and lower-middle-income countries, barriers, global health care, scoping, review

## Abstract

**Background:**

Low- and lower-middle-income countries account for a higher percentage of global epidemics and chronic diseases. In most low- and lower-middle-income countries, there is limited access to health care. The implementation of open-source electronic health records (EHRs) can be understood as a powerful enabler for low- and lower-middle-income countries because it can transform the way health care technology is delivered. Open-source EHRs can enhance health care delivery in low- and lower-middle-income countries by improving the collection, management, and analysis of health data needed to inform health care delivery, policy, and planning. While open-source EHR systems are cost-effective and adaptable, they have not proliferated rapidly in low- and lower-middle-income countries. Implementation barriers slow adoption, with existing research focusing predominantly on technical issues preventing successful implementation.

**Objective:**

This interdisciplinary scoping review aims to provide an overview of contextual barriers affecting the adaptation and implementation of open-source EHR systems in low- and lower-middle-income countries and to identify areas for future research.

**Methods:**

We conducted a scoping literature review following a systematic methodological framework. A total of 7 databases were selected from 3 disciplines: medicine and health sciences, computing, and social sciences. The findings were reported in accordance with the PRISMA-ScR (Preferred Reporting Items for Systematic Reviews and Meta-Analyses extension for Scoping Reviews) checklist. The Mixed Methods Appraisal Tool and the Critical Appraisal Skills Programme checklists were used to assess the quality of relevant studies. Data were collated and summarized, and results were reported qualitatively, adopting a narrative synthesis approach.

**Results:**

This review included 13 studies that examined open-source EHRs’ adaptation and implementation in low- and lower-middle-income countries from 3 interrelated perspectives: socioenvironmental, technological, and organizational barriers. The studies identified key issues such as limited funding, sustainability, organizational and management challenges, infrastructure, data privacy and protection, and ownership. Data protection, confidentiality, ownership, and ethics emerged as important issues, often overshadowed by technical processes.

**Conclusions:**

While open-source EHRs have the potential to enhance health care delivery in low- and lower-middle-income-country settings, implementation is fraught with difficulty. This scoping review shows that depending on the adopted perspective to implementation, different implementation barriers come into view. A dominant focus on technology distracts from socioenvironmental and organizational barriers impacting the proliferation of open-source EHRs. The role of local implementing organizations in addressing implementation barriers in low- and lower-middle-income countries remains unclear. A holistic understanding of implementers’ experiences of implementation processes is needed. This could help characterize and solve implementation problems, including those related to ethics and the management of data protection. Nevertheless, this scoping review provides a meaningful contribution to the global health informatics discipline.

## Introduction

### Background

Low- and lower-middle-income countries (LMICs) are challenging contexts that lack robust infrastructure, technical expertise, and other key resources [1-3]. In most LMICs, there is limited access to information about the health of individuals considered vulnerable, making it difficult to improve health care systems because these settings often require additional funding and maintenance support [4,5]. Furthermore, individuals considered vulnerable seldom have a platform to express their views on health care development and implementation strategies within their communities [6-8]. The resource-scarce settings of LMICs account for a higher percentage of global epidemics and chronic diseases in comparison to the Global North because of limited access to health care, particularly for individuals considered vulnerable [9-11]. This persistent problem of limited access to health care exacerbates inequalities in LMIC settings [12,13], and this calls for innovative and sustainable interventions.

Open-source electronic health records (EHRs) can enhance health care delivery in LMICs by improving the collection, management, and analysis of health data needed to inform health care delivery, policy, and planning. Open source is broadly defined as free software that includes a flexible source code [14-16] that can be modified for various settings [17]. An EHR system is a computerized version of a patient’s (longitudinal) medical records maintained by a given health care provider [18-20]. There is demand for open-source EHR systems in LMICs because they provide implementers with the flexibility to customize the system to meet context-specific needs [21]. An implementer is a member of a local implementing organization who understands the technology, context, and end users. An implementing organization can be a social enterprise or a nongovernment organization that is concerned with the implementation of software. Local implementers and software developers implement open-source EHRs to enhance the delivery of health care for local health facilities [22-25].

The process of implementing open-source EHRs often requires implementing organizations to systematize and conduct an initial analysis of the context, reinvent the software to meet local requirements, lead configuration and installation, and provide user training [4,18,22]. While open-source EHR systems are cost-effective and adaptable, they have not proliferated rapidly in LMICs. Implementation barriers slow adoption, with existing research focusing predominantly on technical issues preventing successful implementation. This scoping review provides an overview of barriers impacting the implementation of open-source EHR systems in LMICs, identifies gaps in the existing literature, and points to opportunities for future research.

### Digital Transformation of Global Health Care

The transformation and innovation of technology over the last few decades have shaped how the global health care industry operates [26]. The demand for universal access to quality health care is rising, putting pressure on governments to develop sustainable solutions for the effective delivery of health care [27]. While digital technologies have the potential to enable equal delivery of better health care [28], they also raise important questions about ethics and data protection [9,29-31], particularly in settings characterized by stark power imbalances. A focus on the development of better technical solutions can distract from the underlying values and ethical concerns associated with health care technologies.

Goal 3 of the United Nations sustainable development goals, “good health and wellbeing,” highlights the importance of improving access to quality health care and managing global health risks [32]. The World Health Organization advocates the use of electronic health tools [33] to enhance the monitoring of patient care [34,35]. An EHR is “a computer based patient records system designed mainly for the use of doctors [or other clinicians that have direct contact with the patient]” [19]. EHR systems can help ease the burden of existing paper record processes and provide better management of patient care electronically [24]. The sustainable development and implementation of EHR systems is a challenge in the contemporary environment [21,36], specifically from political, economic, social, technological, and ethical perspectives [19,37]. Data ownership, informed consent, data protection, and confidentiality are concerns that influence the implementation of many health technologies, including EHR systems [31,38]. Evans [39] and Manders-Huits [40] assert the importance of acknowledging and integrating human values responsibly in health care technology. Therefore, if the context of implementing EHR systems is better understood, it could help address key challenges and barriers from a comprehensive perspective.

### Evolution of Open-Source EHR Systems

There has been a rapid growth of open-source software, notably in the health care sector [18,41]. The phrase “open-source software” was coined in 1998 [17]. Open-source software includes an adaptable source code; when the source code is made publicly accessible under a free license [42], it can be customized by health care providers to meet context-specific requirements [14,15,43,44]. There are various open-source software solutions used for distinct purposes in LMIC settings [45,46], such as the Open Enterprise-level Laboratory Information System (OpenELIS Foundation); District Health Information Software 2 (HISP Centre at the University of Oslo), a web-based platform communicating health data across several levels of a given health care system; and GNU Health, a hospital information system [24,47-49].

An open-source EHR system provides an adaptable and digitalized version of a patient’s medical history, a record that comprises identifiable and personal health information such as demographics, allergies, medication, medical episodes, and health facility visits [18-20]. Open-source EHR systems can enhance health care delivery, inform the development and delivery of health care at the policy level, and lower costs for LMIC settings [21-23,50,51]. A good example of an open-source EHR system, adopted primarily for LMIC settings, is the Open Medical Record System (OpenMRS; OpenMRS Inc) platform [52-55]. The OpenMRS platform is perceived as a collaborative project [25,56-58] aiming to serve a moral purpose by “bringing people together to write code and save lives” [59].

Two early examples of open-source EHRs were the Computer Stored Ambulatory Record and the Veterans Health Information Systems and Technology Architecture, both developed nearly 5 decades ago in a high-income country (the United States) [20,41,60,61]. Since then, there has been an increasing interest in developing and using open-source EHR solutions [17,18,24]. Today, open-source EHR systems are used in many countries, but predominantly in LMICs, where they are seen as helping to address the high cost and inflexibility associated with proprietary EHR systems [4,23,62-65]. Open-source EHR systems can, however, introduce different tensions when implemented in LMIC settings [15,18]. Despite the promises of open-source EHR systems, they are not proliferating as expected. It remains unclear what barriers inhibit their implementation, whether these barriers vary according to different contexts, and how they can be addressed.

### Open-Source Software Versus Proprietary Software

Open-source software offers publicly available source code that can be modified and distributed without incurring licensing fees [14,42,66]. By contrast, proprietary software has copyright restrictions on source code that is not publicly available [16]. Proprietary software such as Microsoft Windows or Office can be perceived as an out-of-the-box solution, and any adaptations required must be completed by the proprietor of the software, resulting in additional fees [17]. Open-source software can be tailored to the specifics of a given context, but this often requires technical expertise and adequate funding for implementation [29,67-69]. Moreover, Reynolds and Wyatt [15] contend that opening the source code compels developers to carefully examine and craft the quality of their code, making bug patching easier, which strengthens the security aspect of open-source systems [41,43]. Proprietary software can be more costly to develop in comparison to open-source software [51], where the source code can be adapted and shared, particularly if there is a need to customize certain system aspects or add additional system features [44,70].

Open-source software does not miraculously address the inadequacies of existing health care systems [17,66,68,71-73]. It still requires a level of expertise and human competence for software design and developing effective systems for end users [74]. Nevertheless, open-source software offers the potential for communities to collaborate effectively, build stronger networks, develop new skills, and transform policy and practice where required [75,76]. Therefore, there are several benefits of using open-source software for health care in LMIC settings [22,51,77].

Open-source EHR systems provide implementers with greater flexibility in building customized systems for a given context and can ease suffering from vendor lock-in often found with proprietary EHR systems [16,44,65,69,77]. For example, vendors of proprietary EHR systems are restrictive in what they share with users, adopt surveillance measures, and impose upgrades [14,15], thus inhibiting freedom and flexibility for end users [18,61]. Conversely, a perceived challenge with open-source EHR systems is that they require specialized skills for implementation and maintenance support [4,17]. Nevertheless, open-source EHR systems allow implementers to adapt a given system to meet context-specific needs [51,70,78].

### Context-Specific Barriers

For LMIC settings, the adoption of open-source EHR systems requires context-specific adaptations [4].

Context can be defined as “the place where an intervention is delivered...or unique factors surrounding an implementation effort.” [79]. There is extensive literature on EHR implementations [4,24,28]; however, existing research has focused on technical perspectives [30,31,40] and factors such as technology infrastructure, power supply, and backups as well as a lack of financial resources [22,65,70,80,81]. However, local and regional context-specific barriers inhibiting the adaptation and implementation of open-source EHRs and issues inhibiting implementing organizations from adopting such technology in LMICs have not yet been researched.

IT implementation requires diverse stakeholders “taking a design and translating it into a working system” [19]. The process of implementing an open-source EHR system involves different stakeholders conducting initial analysis and adapting the software to local requirements, software development, configuration, and installation, as well as providing user training and support [22,82,83]. Open-source EHR implementation requires stakeholder engagement and participation to cocreate a solution that results in a change that generates true value for a given context [79]. Consequently, there is a need for various stakeholders, such as local software developers, implementers, IT providers, and health care practitioners, to work collaboratively when designing and adopting open-source EHR systems for LMIC settings [21,39,84].

Therefore, this scoping review provides an overview of the context-specific barriers and facilitators impacting the adaptation and implementation of open-source EHR systems in LMIC settings. No previous scoping review has explored the perceived contextual barriers impacting the adaptation and implementation of open-source EHRs for LMICs.

## Methods

### Overview

This scoping review aims to provide an overview of the contextual barriers impacting the adaptation and implementation of open-source EHR systems in LMIC settings and outline opportunities for future research. A scoping review methodology was chosen because it provides an understanding of the potential breadth of literature available, lends itself to the identification of relevant concepts and research gaps, and enables the researcher to assess whether a full systematic review is needed or indeed possible [85,86]. The following subsections describe the methodological framework and approach used to undertake the scoping review.

### Stage 1: Identifying the Research Question

To address the interdisciplinary nature and scope of the gap in our understanding, a broad review question was chosen to map the breadth of literature available and identify key concepts and related themes for further exploration. The following research question was formulated: “What are the perceived key contextual barriers impacting the adaptation and implementation of open-source EHRs in LMICs?”

This review follows the 5 stages of the systematic methodological framework for conducting scoping studies by Arksey and O’Malley [87] and follows relevant guidance from the JBI [88], Levac et al [89], and Davis et al [90].

### Stage 2: Identifying Relevant Studies

The 7 most relevant electronic databases were searched from January 1960 to September 2021 (Textbox 1), as EHR developments initially started in the early 1960s [20]. The first reviewer (SB) performed a comprehensive search, which was supported using a population, intervention, and outcome framework [91] that identified four key terms: (1) LMIC, (2) open source, (3) EHR, and (4) adaptation and implementation. The key terms and synonyms are detailed in Multimedia Appendix 1. The selection of databases covered literature from medicine and health sciences, computing, and social sciences. In addition, Open Grey was used to identify relevant gray literature using the key terms, but no results were retrieved. A backward and forward citation search was performed using Google Scholar from reference lists of selected papers to ensure potentially relevant articles were not overlooked [91]. The searches concluded on September 27, 2021. The searches for MEDLINE and MEDLINE In-Process, CINAHL, and Web of Science are included in Multimedia Appendix 2.

**Literature search.**

**Electronic databases**

- MEDLINE and MEDLINE In-Process
- Embase
- CINAHL
- EBSCO Business Source Premier
- Web of Science
- Cochrane Library
- IEEE Xplore

### Stage 3: Study Selection

Studies were included in the review if they were published in English, presented empirical data (including systematic reviews), or nonempirical accounts of experiences and system descriptions on the adaptation and implementation of open-source EHRs in all variations (associated synonyms and phrases) in LMICs. No restrictions were placed on the study design or the format of the publication, and both published and unpublished (gray) literature were included. Papers were reviewed at the title, abstract, and full-text level to exclude articles that did not address the adaptation and implementation of open-source EHRs or were not concerned with LMICs. No other criteria were applied; this was to maximize the search results for an initial inquiry. Multimedia Appendix 3 [3] shows the list of LMICs used for the inclusion criteria.

The following terms were used in the search for relevant material: LMIC, open source, EHR, adaptation, and implementation. These were defined before the search to ensure consistency across the range of databases. The definitions of these key terms can vary; however, for this review, the following definitions were adopted: the World Bank’s definition of LMICs was used [3]; an *EHR* system is a computerized version of a patient’s (longitudinal) medical records, specifically designed for clinicians who have direct contact with patients [19,20]; *open-source software* includes publicly available source code that can be adapted to meet context-specific requirements without incurring any license fees [14-17,42,43]; *implementation* is a complex process that requires organized and deliberated effort to put a given innovation or intervention into practice in such a way that it results in better outcomes for an identified context [79,92]; and *adaptation* is an element of the implementation process. It is the construction of different processes (eg, analysis, customization, installation and configuration, training, and support) that support a given innovation or intervention [93].

**Figure 1:**
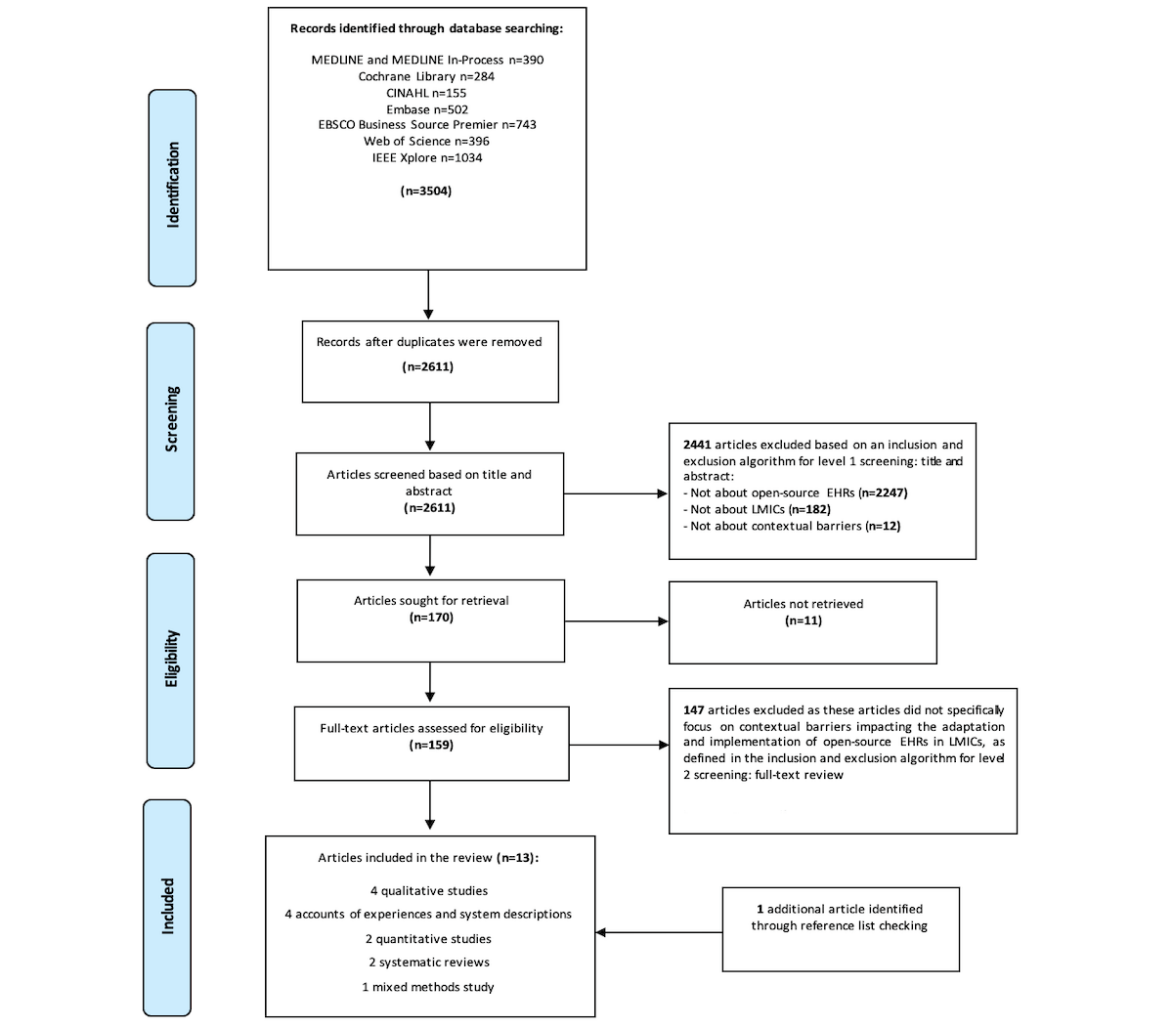
PRISMA-ScR (Preferred Reporting Items for Systematic Reviews and Meta-Analyses extension for Scoping Reviews) flow diagram detailing the study selection process. EHR: electronic health record; LMIC: low- and lower middle–income country

Overall, the search yielded 3504 articles, which were exported into EndNote X9 reference software (Clarivate). After removing 893 (25.49%) duplicates from the 3504 articles, the first reviewer (SB) screened articles via a 2-level process: 2611 (74.51%) titles and abstracts were screened for relevance. Thus, of the 2611 studies, 170 (6.51%) full-text articles were included for review. In addition, a team of 3 reviewers (RR, LJ, and OJ) independently screened 10% (17/170) of the articles to ensure consistency using the inclusion and exclusion algorithm for screening titles and abstracts and then full-text reviews (Multimedia Appendix 4). Any disagreements were discussed and resolved among the reviewers. Finally, 12 studies met all criteria of the inclusion and exclusion algorithm. An additional article was discovered through the forward and backward reference list checks. In total, 13 studies were found to satisfy the inclusion and exclusion criteria and were included in the review. The PRISMA-ScR (Preferred Reporting Items for Systematic Reviews and Meta-Analyses extension for Scoping Reviews) [94] flow diagram was used to report the results of the study selection process (Figure 1).

### Stage 4: Charting the Data

A standard data extraction form was used to obtain an overview of the 13 selected studies. For each study, the following information was extracted: authors publication year, country of origin (where the study was conducted), aims or purpose, study design, study population and sample size (if applicable), methods, intervention type (open-source EHRs), and key findings that relate to the scoping review question. Any inconsistencies were discussed and resolved among the reviewers.

### Stage 5: Collating, Summarizing, and Reporting the Results

After charting the key data from the 13 studies, a qualitative thematic analysis [95,96] and a synthesis approach was adopted [89]. The first reviewer coded, categorized, and grouped the results into key themes to address the scoping review question and identify the implications for future research. The thematic analysis was inspired by existing frameworks presented by Jawhari et al [84] and Muinga et al [21], who synthesized key findings using relevant categories to illustrate the advantages and barriers to implementing electronic medical record systems in a given geographically bounded space. However, this scoping review focused on the global level, examining contextual barriers impacting the adaptation and implementation of open-source EHR systems for LMIC settings (local and regional). Therefore, the categories and key themes identified in this review were generated from the analysis of the 13 studies.

### Quality Assessment

The Mixed Methods Appraisal Tool and the Critical Appraisal Skills Programme checklists were adopted to assess the quality, where relevant, of the included studies, as the review comprises a broad range of study designs and methodologies [97-99].

## Results

### Characteristics of Studies

Table 1 briefly summarizes the characteristics of the included studies. All studies were published between 2002 and 2021. Of the 13 studies included in this review, geographically, 9 (69%) report research conducted in Sub-Saharan Africa, Uganda, Ethiopia, Kenya, Ghana, and Sierra Leone [9,21,24,28,44,84,100-102]. A total of 2 studies were conducted in South Asia: India and Nepal [103,104]. The type of research varied across the spectrum, with the most common following a qualitative design, with the use of interviews, surveys including qualitative questions, participatory techniques and observations used to address the design, and barriers and facilitators with implementing open-source EHRs [9,21,84,101].

**Table 1:** A summary of the characteristics of the included studies.

Study and country	Study design	Urban or rural
Mohammed-Rajput et al [9], 2011; Kenya, Rwanda, Lesotho, Tanzania, Uganda, and Malawi	Qualitative	Rural
Syzdykova et al [24], 2017; Ethiopia	Systematic review	Rural
Muinga et al [21], 2018; Kenya	Qualitative	Rural
Oza et al [28], 2017; Sierra Leone	Quantitative	Rural
Akanbi et al [44], 2012; Sub-Saharan Africa	Systematic review	Rural
Fish and Guha [68], 2020; Haiti	Descriptive	Rural
Verma et al [83], 2021; Kenya, Nepal, Liberia, Lesotho, Haiti, Uganda, Sierra Leone, Rwanda, Nigeria, Mozambique, Malawi, Kazakhstan, India, Ethiopia, Democratic People’s Republic of Korea, and Peru	Mixed methods	Rural
Jawhari et al [84], 2016; Kenya	Qualitative	Urban
Gainer et al [100], 2012; Ethiopia	Descriptive	Rural
Gyamfi et al [101], 2017; Ghana	Qualitative	Urban
Were et al [102], 2010; Uganda	Quantitative	Urban
Anantraman et al [103], 2002; India	Descriptive	Rural
Raut et al [104], 2017; Nepal	Descriptive	Rural

Of the 2 quantitative studies, one used controlled observations of clinicians and patients before implementation and postimplementation and the other used a survey with standardized measures [28,102]. One study adopted a mixed methods design inclusive of quantitative and qualitative aspects [83]. The systematic reviews looked at various open-source EHRs in LMICs and the challenges inhibiting implementation [24,44]. Other studies were more accounts of experiences and system descriptions [68,100,103,104]. Most of the studies (10/13, 77%) addressed rural areas [9,21,24,28,44,68,83,100,103,104], and other studies (3/13, 23%) addressed urban settings [84,101,102]. Multimedia Appendix 5 [9,21,24,28,44,68,83,84,100-104] illustrates the detailed characteristics of each included study.

### Quality Assessment

The Mixed Methods Appraisal Tool checklist was used for 7 studies [9,21,28,83,84,101,102], and the Critical Appraisal Skills Programme Systematic Review checklist was used for 2 systematic reviews [24,44]. A total of 4 studies [68,100,103,104] were nonempirical accounts of experiences and system descriptions of open-source EHR implementations, which provided interesting insights but were not suitable for quality assessment. In total, 2 qualitative studies in this review were of moderate quality, as it is unclear what data were included in the analysis [9], and there is no information about the analysis [21]. A total of 5 studies were assessed as good quality: 2 qualitative [84,101], 2 quantitative [28,102], and the mixed methods study [83]. Moreover, 2 systematic reviews were assessed as moderate quality [24,44] with less scientific rigor, as there was no information about the quality of the included studies. The data table reporting the quality assessments is included in Multimedia Appendix 6 [9,21,24,28,44,83,84,101,102].

### Contextual Barriers Impacting Open-Source EHR Implementations

#### Overview

The 13 included studies provide a broad overview of the perceived contextual barriers impacting the adaptation and implementation of open-source EHRs in LMICs. Three distinct but interrelated perspectives emerged from the thematic review: (1) *socioenvironmental barriers* draw attention to issues surrounding the relationship between humans in a given society and their external environments, such as social capital, social cohesion, infrastructure (local and regional), culture, values, languages, institutions, and stakeholders; (2) *technological barriers* emphasize the issues surrounding the software and hardware used in open-source EHR implementations; and (3) *organizational barriers* draw attention to the operational practices in organizational structures of open-source EHR implementations. In Textbox 2, we organize our findings around these 3 themes. While some issues can be assigned to one thematic barrier, others are cross-cutting barriers, as summarized in Textbox 2. Some subcategories are addressed within >1 barrier, where they are interpreted through different lenses. For example, issues such as infrastructure, ethical practices, or finance are not only of importance from a socioenvironmental perspective but are also raised, albeit in different ways, by researchers who adopt a technological or organizational perspective.

**Thematic framework (contextual barriers and subcategories [subsumed issues]) for the analysis of adaptation and implementation barriers impacting open-source electronic health record systems in low- and lower-middle-income countries.**

**Socioenvironmental barriers**

- Lack of social cohesion: voice and trust [9,21,24,28,44,84,100,102-104]
- Require stakeholder engagement and participation as well as political support [9,21,24,28,44,68,83,84,100-104]
- Sustainability: co-design and collaboration [9,21,24,28,44,68,83,84,100-104]
- Social capital and lack of funding [9,21,24,28,44,68,83,84,100-104]
- Language barriers: reliance on the local language [44,68]
- Epidemic diseases (health emergencies) [9,21,28,44,68,84,100,103,104]
- Environment—lack of resources (poverty) [9,21,24,28,44,68,83,84,100-104]
- Infrastructure: access to electricity, local network coverage, medical facilities, and rural and urban health [9,21,24,28,44,68,83,84,100-104]

**Technological barriers**

- Infrastructure (power, network, and technology) [9,21,24,28,44,68,83,84,100,101,104]
- Data security, privacy and confidentiality, storage, quality, and ethics [9,21,24,28,44,68,83,84,100-102,104]
- Software and hardware suitability (context specific) [9,21,28,44,68,84,100-102,104]
- Interoperability [21,24,28,68,84,100]
- Sustainability of systems [9,21,28,44,68,83,84,100-102,104]
- User interface: not supporting different clinical roles [9,21,24,28,44,68,83,84,100,101]
- Patient-centered design (lack of end user [clinicians, health facility administrators, and patients] involvement) [9,21,28,68,83,84,100,103,104]

**Organizational barriers**

- Finance and benefactors (costs for context-specific implementations and maintenance) [9,21,24,28,44,68,83,84,100,101,103,104]
- Human resource development (training, IT skills or expertise, support, and staff shortage) [9,21,24,28,44,68,83,84,100-102,104]
- Resistance to change [21,28,44,68,83,84,100,101]
- Organizational culture and change management [9,21,24,28,44,68,83,84,100,101]
- Strategic, agile planning (context specific) [9,21,24,28,68,84,100,101,104]
- Lack of leadership [21,24,28,44,68,83,84,101,104]
- Documentation and resources [9,24,28,68,83,84,100,101,104]
- Self-sufficiency [9,21,68,84,101]
- Communication and transparency [9,21,24,28,83,84,100,101,103]
- Trust, social cohesion, and ownership [9,21,68,83,84,101,104]
- Workflow pressure, staff morale, and ethical practices [9,21,24,28,44,84,100-102,104]
- More reactive than proactive [9,21,28,44,68,84,101,103]
- Deep-rooted habits (relative to culture or specific context) [28,44,68,83,84,100,101]

Adaptation of open-source EHR software is needed because of contextual factors such as limited resources, infrastructure, organizational setup, and workflows. LMIC settings have unique requirements. There are different user realities to consider, and specific issues seem to determine the success or failure of open-source EHR implementations [105]. These contextual barriers have been found to include political, economic, social, and technological issues [31,106]. To better understand these barriers to implementing open-source EHRs in LMICs, one has to first acknowledge the given setting, understand end-user realities, and consider the availability of resources [107].

#### Socioenvironmental Barriers Impacting Open-Source EHR Adaptation and Implementation

The socioenvironmental barriers include diverse issues: resource scarcity, limited political support, and socioeconomic difficulties. These issues impact open-source EHR adaptation and implementation in LMICs. Nearly all studies [9,21,24,28,44,68,83,84,100,102-104] show that a lack of resources, such as technical expertise, power and connectivity, investment, workstations, human resource, and support, impedes successful adaptations and implementations.

The lack of effective stakeholder engagement and participation may impact long-term sustainability and health care delivery in LMICs [102]. Anantraman et al [103] state that the adaptation and implementation of open-source EHRs are best supported through the effective involvement of various stakeholders, such as end users, IT providers, clinicians, and developers [21]. Moreover, Akanbi et al [44], Syzdykova et al [24], and Muinga et al [21] highlighted the importance of government intervention in supporting open-source EHR projects, both before implementation and postimplementation. This is confirmed by Raut et al [104]. They describe the success of 2 open-source EHR implementations in rural Nepal, primarily because of the commitment, cohesion, and support provided by the government of Nepal (at the local and regional level) as a key stakeholder.

Jawhari et al [84] reported the importance of addressing social challenges and health inequalities within communities considered marginalized in urban-poor contexts as a key issue, but it is one that is often overlooked in adaptation and implementation. Moreover, how implementers enact the implementation process and how they respond to the perceived challenges in local settings remain unclear. An important finding by Jawhari et al [84] illustrates that socially constructed stigmas associated culturally with certain diseases can impact the acceptance and effective use of open-source EHRs in slums and other urban-poor settings, where some patients use multiple identities or show resistance to their data being recorded electronically. Resistance from patients may be related to “general mistrust of systems that might track identities” [84], as often these individuals reside in insecure tenures and lack rights and legal status. These issues are perceived to be influenced simultaneously by the *technological barriers*, such as poor patient-centered design and data privacy and confidentiality issues, and *organizational barriers*, such as lack of trust, communication issues, limited funds for context-specific implementations, and maintenance support [68].

#### Technological Barriers Impacting Open-Source EHR Adaptation and Implementation

Our review suggests that technological barriers include power outages, network failure, interoperability, hardware suitability, data privacy, and system sustainability [9,68,83,100]. Data security, privacy, and confidentiality issues emerged as a critical need in terms of addressing adequate security features before adaptation and implementation, with concerns that patient data are too broadly accessible to health care professionals not directly involved in a patient’s care [21,84,101].

Several studies [9,21,24,28,44,84,100,101,103,104] reported on the limited interoperability of their open-source EHRs with legacy health systems and how that can hinder successful adaptations and implementations. These are additionally influenced by socioenvironmental (lack of resources and stakeholder intervention) and organizational (lack of expertise and finance) barriers [68]. Unreliable infrastructure at local and regional levels is perceived to be a major challenge encountered in urban and rural settings, often because of limited funding, poor stakeholder intervention, and limitations of key resources [83,101], issues that are perceived to be influenced by socioenvironmental and organizational barriers. Moreover, there is little information on how implementers address the identified challenges responsively.

#### Organizational Barriers Impacting Open-Source EHR Adaptation and Implementation

There are broad issues, particularly relating to the management of organizations and human resources, that are categorized within the organizational barriers theme. For example, finance, human resource development, strategic planning, change management, data ownership, social cohesion, trust, and ethical practice influence open-source EHR adaptation and implementation in LMICs. The ethical issues intersect across multiple perspectives. From an organizational perspective, ethical issues comprise ownership, trust, management, and organizational culture [9,21,101]. From a technological perspective, ethical issues raise concerns for data privacy and confidentiality, protection, and infrastructure [24,100]. From a socioenvironmental perspective, the ethical issues emphasize sustainability and context, stakeholder intervention, and socioeconomic factors [68,84].

Several studies identified hybrid interventions where open-source EHR systems were used alongside paper processes and described how this approach appeared to impede successful adaptations and implementations [28,44,84,100]. This may be interpreted in terms of inadequate stakeholder intervention, unreliable local and regional infrastructure, lack of proficiency and training, and inherent habits impeding open-source EHR adaptation and implementation [68,84,104]. The practice of such hybrid interventions lowers the true value of open-source EHR success and constructs a dysfunctional organizational environment in LMIC settings, overburdening staff, increasing workload pressure, hindering morale, and influencing resistance to change [28,83,100]. Furthermore, there is limited information on how exactly open-source EHR systems are adapted and implemented in LMICs and the roles of implementers in the implementation process.

From a socioenvironmental and technological perspective, finance can be seen as an important factor for resources and sustainability. However, from an organizational perspective, finance is perceived to be a major challenge that impacts the adaptation and implementation of open-source EHR systems in LMICs. This can be observed in terms of the inability to source proficient software developers and IT providers, inadequate staff training and support, limited funding from potential stakeholders, and overreliance on funders [9,44,101]. The lack of adequate training and support from stakeholders, such as implementers, developers, IT providers, funders, and government, can adversely influence open-source EHR ownership [21,83]. This is understood in terms of inefficient organizational responsibility of system management, quality data, and conflicting stakeholder relations: the lack of leadership, inability to take responsibility, risking patient data, raising confidentiality concerns, poor communication, and trust leading to an insecure organizational environment [28,68,84,100]. End users report a lack of leadership, motivation, and communication and suggest the need for a reliable organizational culture and human resources that provide adequate support and training [101].

## Discussion

### Principal Findings

This scoping review revealed thematic sets of socioenvironmental, technological, and organizational barriers to the adaptation and implementation of open-source EHR systems in LMICs. Specific issues, which were mentioned often, include organizational and management, limited funding, local and regional infrastructure, data privacy, confidentiality and protection, ownership, and sustainability, which appear to influence the adaptation and implementation of open-source EHRs in LMICs [9,21,83,101]. From an organizational and management perspective, data protection and confidentiality, ownership, and ethics emerge as important issues that are context dependent. The process of implementation is a key topic to explore because it is an issue often overshadowed by technical processes, with less emphasis on the social perspective [30,31,40,68], which requires the application of a relational lens to gain an in-depth understanding [108]. There is a need for a holistic understanding to explore how implementing organizations in LMIC settings addresses socioenvironmental, technical, and organizational barriers.

This scoping review shows that shortfalls in funding, leadership, and organizational and human resources also give rise to serious data protection and confidentiality issues [44,68,84,101]. It has been argued that there is a need for implementing organizations to develop self-sufficiency and take responsibility for data protection and ownership, establish local support and training initiatives, and build relations with key stakeholders for long-term sustainability [102,104]. This notion shows that implementing organizations could better engage with key stakeholders and develop better ethical practices in terms of taking responsibility, managing organizational culture, and implementing change responsibly. Furthermore, the adaptation and implementation of open-source EHR systems can be supported through collaborative actions such as ensuring local sites are sufficiently equipped with access to power, considering data protection and confidentiality, ensuring that networks and workstations are integrated efficiently, and considering alternative measures in case of emergencies [21,28,84]. Nevertheless, strategic and agile organizational planning are also perceived as essential for successful implementations in LMICs.

There is a need to examine the given context; encourage meaningful change; involve end users such as clinicians, health facility administrators, and patients in implementation design; and collaborate with a diverse range of stakeholders cohesively for successful implementations [21,68,84,101]. Key stakeholders, particularly government, need to collaborate, support, and develop sustainable context-specific open-source EHR implementations that offer robust functionality. Unreliable technical infrastructure at the local and regional levels can have negative implications for open-source EHR adaptations and implementations in LMICs. Open-source EHR systems can enable better health care access, have a positive impact on medical record quality, and enhance the potential of patient care [23,77]. However, the challenge with adapting and implementing open-source EHR systems is finding local expertise, technical skills, and sophisticated support for implementation and maintenance, which are key ingredients needed to make it work meaningfully in LMIC settings [4,15,17,83]. Therefore, there is a disconnect between the understanding of the context-specific barriers, the implementation process, and stakeholder relations.

These findings show that, depending on the thematic perspective adopted, different issues stand out. Each of the 3 perspectives brings into view some issues and obscures others. Hence, a holistic view, inclusive of all 3 perspectives, is needed to better understand the implementation process and how challenges are addressed locally. Context-specific barriers and issues in open-source EHR implementations can be better understood through the voices of key stakeholders (implementers and end users) on the ground [21,68,83,101,106].

Some challenges need to be addressed systematically, such as considering the socioenvironmental and organizational perspectives, understanding change from a reflexive perspective, conducting responsible operations, integrating values, and including stakeholders in design before adapting and implementing open-source EHR systems in LMICs [84,103]. For example, Oza et al [28] highlight that adapting and implementing an open-source EHR system during a health emergency, the Ebola outbreak, was a major problem as the outbreak (fortunately) started to decline, which limited the usefulness of the system (postimplementation) in a resource-scarce environment. Furthermore, designing open-source EHR systems while epidemic cases are increasing is not a sustainable action; intervention needs to be planned earlier and in retrospect to address the true value of what open-source EHR systems can offer in health emergencies [28]. This perception shows that different socioenvironmental barriers to adaptation and implementation can arise, depending on the situation. The findings indicate that understanding and collectively addressing the perceived contextual barriers in LMICs before implementation is of paramount importance. There is a gap in our understanding of how open-source EHR systems are implemented and the importance of implementers in the implementation process.

Literature, which was not included in the study selection but was considered useful, provided supplementary information. For example, it indicates the presence of a wider discourse on how stakeholders need to reflect on ownership, data confidentiality, protection of information, patient dignity, and addressing unethical operations [15,30,37,39,63]. Were and Meslin [31] contend that many ethical frameworks exist (relating to issues of research ethics); however, there are no such frameworks that evaluate how well open-source EHR implementations address ethical issues in LMIC settings. While the level of discussion in high-income countries focuses on “privacy, confidentiality, data security, informed consent, data ownership, and secondary use of data” [31], little research has been conducted to address similar ethical issues existing with open-source EHR implementations in LMICs. Therefore, if the open-source EHR implementation process is better understood, it could help implementers address implementation readiness issues effectively and improve outcomes for LMICs.

There is also the issue of being responsible and accountable for data quality and negotiating during implementation. This points to the broader issue of whether responsibility is socially assumed or coconstructed. Manders-Huits [40] highlights that “[health] technologies can promote or undermine specific human values...technology shapes our practices and institutions in important ways.” This shows that developers and implementers, as key stakeholders, have a level of responsibility in acting purposefully in a role to embed human values and ethical considerations within open-source EHR system adaptations and implementations. There is potential for research to explore the roles of local implementing organizations that play a fundamental role in adopting open-source EHRs on the ground.

The findings in this review highlight that more funding is required to achieve better EHR implementation readiness in LMIC settings [9,68,84,101]. There are several eHealth readiness assessment (eHRA) frameworks and related tools within the existing literature that have identified 8 readiness types: organizational, technology and infrastructure, health care providers, engagement, social, core, government, and public or patient [109-111]. The 3 thematic barriers and the subcategories identified in Textbox 2 resonate with some aspects of the 8 eHRA types. However, existing eHRA frameworks are found to be inadequate to support implementation readiness in the context of LMIC settings [112-114]. It is key to better understand the implementation process from a holistic perspective. There are limitations when one looks at adaptation and implementation from one of the 3 perspectives (socioenvironmental, technological, and organizational barriers). Hence, implementation needs to be clearly understood holistically, through implementers’ perspectives, to better navigate barriers encountered in LMICs.

### Strengths and Limitations

The results of this review may be limited because only studies published in English were included, and hence, they are subject to cultural selection bias. Nevertheless, no other restrictions were placed on the study design or the publication format to maximize the search results. The identified key search terms, LMIC, open source, EHR, and adaptation and implementation, were defined before the search to ensure consistency across the range of databases. In addition, using an inclusion and exclusion algorithm to screen titles, abstracts, and full-text reviews helped to ensure consistency. This scoping review identified a small number of helpful studies exploring the contextual barriers impacting open-source EHR implementations in LMICs. However, existing research does not examine how implementers understand and navigate the implementation process more closely and how they respond to barriers in a given context. Therefore, the identified opportunity in the literature highlights a need to conduct further research in this area.

We acknowledge that the use of picture archiving and communication systems in LMIC settings could be a useful area for future research. Furthermore, research opportunities could explore the acceptability of international standards and compare alternatives such as Health Level Seven, Fast Health care Interoperability Resources, and Digital Imaging and Communications in Medicine in LMIC settings. The comparison of open-source EHRs with web applications and mobile apps or focusing evaluation research is also a useful direction for future research.

### Conclusions

Open-source EHRs have the potential to facilitate enhanced health care and encourage sustainable development in LMICs, where designed effectively and responsibly within country-specific requirements. This scoping review provides an overview of the contextual barriers impacting the adaptation and implementation of open-source EHR systems in LMIC settings. It shows that depending on the adopted perspective to implementation, different implementation barriers come into view. A dominant focus on technology distracts from socioenvironmental and organizational barriers impacting the proliferation of open-source EHRs. Each of the 3 perspectives (socioenvironmental, technological, and organizational) draws attention to key implementation issues and highlights the important role implementers may play in addressing these issues. However, by itself, none of these 3 perspectives enable us to appreciate more fully the many interlocking challenges associated with implementing open-source EHRs in LMIC settings.

It is vital to consider the more specific context in which open-source EHRs are to be adopted and to address the need for effective implementation through a better understanding and collaboration with all stakeholders. A lack of empirical evidence limits our understanding of how exactly open-source EHR systems are adapted and implemented. Research is required to explore the roles of local implementing organizations in addressing implementation barriers in LMIC settings. A holistic understanding of implementers’ experiences of implementation processes is needed. This could help characterize and solve implementation problems, including those related to ethics and the management of data protection. Nevertheless, this scoping review provides a meaningful contribution to the global health informatics discipline. We hope that the review results will inform areas for future research and enhance implementation.

## Appendix

Multimedia Appendix 1 Identification of the key terms and synonyms using the population, intervention, and outcome framework.

Multimedia Appendix 2 Search terms and search strategy for the scoping review.

Multimedia Appendix 3 List of low- and lower middle–income countries.

Multimedia Appendix 4 The inclusion and exclusion algorithm for screening articles.

Multimedia Appendix 5 Characteristics of the included studies.

Multimedia Appendix 6 Quality assessment tools table.

Multimedia Appendix 7 Preferred Reporting Items for Systematic Reviews and Meta-Analyses extension for Scoping Reviews (PRISMA-ScR) checklist.

## Abbreviations

EHR: electronic health record
eHRA: eHealth readiness assessment
LMIC: low- and lower-middle-income country
PRISMA-ScR: Preferred Reporting Items for Systematic Reviews and Meta-Analyses extension for Scoping Reviews

## Data Availability

All data generated or analyzed during this study are included in this published article.
