# Supplementary material for: Contextual Barriers to Implementing Open-Source Electronic Health Record Systems for Low- and Lower-Middle-Income Countries: Scoping Review
Source: J Med Internet Res. 2024 Aug 1;26:e45242. doi: 10.2196/45242 (PMC11327637; doi:10.2196/45242)
Supplement: Multimedia Appendix 1 [file jmir_v26i1e45242_app1.docx]

| Identification of key terms and synonyms using PIO framework  Review question: What are the perceived key contextual barriers impacting the adaptation and implementation of open source EHRs in LMICs? | | | | |
| --- | --- | --- | --- | --- |
|  | ***Population*** | ***Intervention*** | | ***Outcome*** |
|  | **LMICs** | **Open source** | **EHR** | **Adaptation and implementation** |
| *Synonyms* | Constrained settings  Developing countries  Developing economies  Developing nations  Developing world  Economically developing countries  Economically developing nations  Emerging countries  Emergent nations  Global south  Informal settlements  Limited resource settings  Low-resource settings  Low- and middle-income countries <add “”>  Poor countries  Poor resource environments  Resource-constrained environments  Resource-limited settings  Resource poor settings  Resource poor environments  Slum settings  Slum populations  Third World  Urban slums  Urban poverty  Underserved environments  Underdeveloped nations  Underprivileged countries | Open source | Computerised patient records (CPR) <add wildcard ‘?’>  County electronic health record (CEHR)  Electronic patient record (EPR)  Electronic medical record (EMR)  Electronic nursing record (ENR)  Medical record system (MRS)  National electronic health record (EHR)  Nationwide electronic health record (NEHR)  Patient record system (PRS)  Patient medical record (PMR)  Personal health record (PHR)  Personally controlled health management systems (PCHMS)  Problem-oriented medical record (POMR)  Source-oriented medical record (SOMR)  Time-oriented medical record | Acceptance  Adoption  Alteration  Fitting  Integration  Modification  Usage |
