# Supplementary material for: Contextual Barriers to Implementing Open-Source Electronic Health Record Systems for Low- and Lower-Middle-Income Countries: Scoping Review
Source: J Med Internet Res. 2024 Aug 1;26:e45242. doi: 10.2196/45242 (PMC11327637; doi:10.2196/45242)
Supplement: Multimedia Appendix 2 [file jmir_v26i1e45242_app2.docx]

**Search terms and search strategy for scoping review:**

| **MEDLINE and MEDLINE In Process** | |
| --- | --- |
| No. | **Searches** |
| 1 | exp Developing Countries/ or Global Health/ |
| 2 | LMIC*.mp. [mp=title, abstract, original title, name of substance word, subject heading word, floating sub-heading word, keyword heading word, organism supplementary concept word, protocol supplementary concept word, rare disease supplementary concept word, unique identifier, synonyms] |
| 3 | Constrained setting*.mp. [mp=title, abstract, original title, name of substance word, subject heading word, floating sub-heading word, keyword heading word, organism supplementary concept word, protocol supplementary concept word, rare disease supplementary concept word, unique identifier, synonyms] |
| 4 | Developing countr*.mp. [mp=title, abstract, original title, name of substance word, subject heading word, floating sub-heading word, keyword heading word, organism supplementary concept word, protocol supplementary concept word, rare disease supplementary concept word, unique identifier, synonyms] |
| 5 | Developing econom*.mp. [mp=title, abstract, original title, name of substance word, subject heading word, floating sub-heading word, keyword heading word, organism supplementary concept word, protocol supplementary concept word, rare disease supplementary concept word, unique identifier, synonyms] |
| 6 | Developing nation*.mp. [mp=title, abstract, original title, name of substance word, subject heading word, floating sub-heading word, keyword heading word, organism supplementary concept word, protocol supplementary concept word, rare disease supplementary concept word, unique identifier, synonyms] |
| 7 | Developing world.mp. [mp=title, abstract, original title, name of substance word, subject heading word, floating sub-heading word, keyword heading word, organism supplementary concept word, protocol supplementary concept word, rare disease supplementary concept word, unique identifier, synonyms] |
| 8 | Economically developing countr*.mp. [mp=title, abstract, original title, name of substance word, subject heading word, floating sub-heading word, keyword heading word, organism supplementary concept word, protocol supplementary concept word, rare disease supplementary concept word, unique identifier, synonyms] |
| 9 | Economically developing nation*.mp. [mp=title, abstract, original title, name of substance word, subject heading word, floating sub-heading word, keyword heading word, organism supplementary concept word, protocol supplementary concept word, rare disease supplementary concept word, unique identifier, synonyms] |
| 10 | Emerging countr*.mp. [mp=title, abstract, original title, name of substance word, subject heading word, floating sub-heading word, keyword heading word, organism supplementary concept word, protocol supplementary concept word, rare disease supplementary concept word, unique identifier, synonyms] |
| 11 | Emergent nation*.mp. [mp=title, abstract, original title, name of substance word, subject heading word, floating sub-heading word, keyword heading word, organism supplementary concept word, protocol supplementary concept word, rare disease supplementary concept word, unique identifier, synonyms] |
| 12 | Global south.mp. [mp=title, abstract, original title, name of substance word, subject heading word, floating sub-heading word, keyword heading word, organism supplementary concept word, protocol supplementary concept word, rare disease supplementary concept word, unique identifier, synonyms] |
| 13 | Informal settlement*.mp. [mp=title, abstract, original title, name of substance word, subject heading word, floating sub-heading word, keyword heading word, organism supplementary concept word, protocol supplementary concept word, rare disease supplementary concept word, unique identifier, synonyms] |
| 14 | Limited resource setting*.mp. [mp=title, abstract, original title, name of substance word, subject heading word, floating sub-heading word, keyword heading word, organism supplementary concept word, protocol supplementary concept word, rare disease supplementary concept word, unique identifier, synonyms] |
| 15 | Low-resource setting*.mp. [mp=title, abstract, original title, name of substance word, subject heading word, floating sub-heading word, keyword heading word, organism supplementary concept word, protocol supplementary concept word, rare disease supplementary concept word, unique identifier, synonyms] |
| 16 | "Low and middle income countr*".mp. [mp=title, abstract, original title, name of substance word, subject heading word, floating sub-heading word, keyword heading word, organism supplementary concept word, protocol supplementary concept word, rare disease supplementary concept word, unique identifier, synonyms] |
| 17 | Poor countr*.mp. [mp=title, abstract, original title, name of substance word, subject heading word, floating sub-heading word, keyword heading word, organism supplementary concept word, protocol supplementary concept word, rare disease supplementary concept word, unique identifier, synonyms] |
| 18 | Poor resource environment*.mp. [mp=title, abstract, original title, name of substance word, subject heading word, floating sub-heading word, keyword heading word, organism supplementary concept word, protocol supplementary concept word, rare disease supplementary concept word, unique identifier, synonyms] |
| 19 | Resource-constrained environment*.mp. [mp=title, abstract, original title, name of substance word, subject heading word, floating sub-heading word, keyword heading word, organism supplementary concept word, protocol supplementary concept word, rare disease supplementary concept word, unique identifier, synonyms] |
| 20 | Resource-limited setting*.mp. [mp=title, abstract, original title, name of substance word, subject heading word, floating sub-heading word, keyword heading word, organism supplementary concept word, protocol supplementary concept word, rare disease supplementary concept word, unique identifier, synonyms] |
| 21 | Resource poor setting*.mp. [mp=title, abstract, original title, name of substance word, subject heading word, floating sub-heading word, keyword heading word, organism supplementary concept word, protocol supplementary concept word, rare disease supplementary concept word, unique identifier, synonyms] |
| 22 | Resource poor environment*.mp. [mp=title, abstract, original title, name of substance word, subject heading word, floating sub-heading word, keyword heading word, organism supplementary concept word, protocol supplementary concept word, rare disease supplementary concept word, unique identifier, synonyms] |
| 23 | Slum setting*.mp. [mp=title, abstract, original title, name of substance word, subject heading word, floating sub-heading word, keyword heading word, organism supplementary concept word, protocol supplementary concept word, rare disease supplementary concept word, unique identifier, synonyms] |
| 24 | Slum population*.mp. [mp=title, abstract, original title, name of substance word, subject heading word, floating sub-heading word, keyword heading word, organism supplementary concept word, protocol supplementary concept word, rare disease supplementary concept word, unique identifier, synonyms] |
| 25 | Third World.mp. [mp=title, abstract, original title, name of substance word, subject heading word, floating sub-heading word, keyword heading word, organism supplementary concept word, protocol supplementary concept word, rare disease supplementary concept word, unique identifier, synonyms] |
| 26 | Urban slum*.mp. [mp=title, abstract, original title, name of substance word, subject heading word, floating sub-heading word, keyword heading word, organism supplementary concept word, protocol supplementary concept word, rare disease supplementary concept word, unique identifier, synonyms] |
| 27 | Urban poverty.mp. [mp=title, abstract, original title, name of substance word, subject heading word, floating sub-heading word, keyword heading word, organism supplementary concept word, protocol supplementary concept word, rare disease supplementary concept word, unique identifier, synonyms] |
| 28 | Underserved environment*.mp. [mp=title, abstract, original title, name of substance word, subject heading word, floating sub-heading word, keyword heading word, organism supplementary concept word, protocol supplementary concept word, rare disease supplementary concept word, unique identifier, synonyms] |
| 29 | Underdeveloped nation*.mp. [mp=title, abstract, original title, name of substance word, subject heading word, floating sub-heading word, keyword heading word, organism supplementary concept word, protocol supplementary concept word, rare disease supplementary concept word, unique identifier, synonyms] |
| 30 | Underprivileged countr*.mp. [mp=title, abstract, original title, name of substance word, subject heading word, floating sub-heading word, keyword heading word, organism supplementary concept word, protocol supplementary concept word, rare disease supplementary concept word, unique identifier, synonyms] |
| 31 | 1 or 2 or 3 or 4 or 5 or 6 or 7 or 8 or 9 or 10 or 11 or 12 or 13 or 14 or 15 or 16 or 17 or 18 or 19 or 20 or 21 or 22 or 23 or 24 or 25 or 26 or 27 or 28 or 29 or 30 |
| 32 | medical records/ or health records, personal/ or medical records, problem-oriented/ or exp medical records systems, computerized/ or exp electronic health records/ or exp health information exchange/ |
| 33 | (Computeri?ed patient record* or CPR).mp. [mp=title, abstract, original title, name of substance word, subject heading word, floating sub-heading word, keyword heading word, organism supplementary concept word, protocol supplementary concept word, rare disease supplementary concept word, unique identifier, synonyms] |
| 34 | (County electronic health record* or CEHR*).mp. [mp=title, abstract, original title, name of substance word, subject heading word, floating sub-heading word, keyword heading word, organism supplementary concept word, protocol supplementary concept word, rare disease supplementary concept word, unique identifier, synonyms] |
| 35 | (Electronic patient record* or EPR*).mp. [mp=title, abstract, original title, name of substance word, subject heading word, floating sub-heading word, keyword heading word, organism supplementary concept word, protocol supplementary concept word, rare disease supplementary concept word, unique identifier, synonyms] |
| 36 | (Electronic medical record* or EMR*).mp. [mp=title, abstract, original title, name of substance word, subject heading word, floating sub-heading word, keyword heading word, organism supplementary concept word, protocol supplementary concept word, rare disease supplementary concept word, unique identifier, synonyms] |
| 37 | (Electronic nursing record* or ENR*).mp. [mp=title, abstract, original title, name of substance word, subject heading word, floating sub-heading word, keyword heading word, organism supplementary concept word, protocol supplementary concept word, rare disease supplementary concept word, unique identifier, synonyms] |
| 38 | (Medical record system* or MRS*).mp. [mp=title, abstract, original title, name of substance word, subject heading word, floating sub-heading word, keyword heading word, organism supplementary concept word, protocol supplementary concept word, rare disease supplementary concept word, unique identifier, synonyms] |
| 39 | (National electronic health record* or National EHR*).mp. [mp=title, abstract, original title, name of substance word, subject heading word, floating sub-heading word, keyword heading word, organism supplementary concept word, protocol supplementary concept word, rare disease supplementary concept word, unique identifier, synonyms] |
| 40 | (Nationwide electronic health record* or NEHR*).mp. [mp=title, abstract, original title, name of substance word, subject heading word, floating sub-heading word, keyword heading word, organism supplementary concept word, protocol supplementary concept word, rare disease supplementary concept word, unique identifier, synonyms] |
| 41 | (Patient record system* or PRS*).mp. [mp=title, abstract, original title, name of substance word, subject heading word, floating sub-heading word, keyword heading word, organism supplementary concept word, protocol supplementary concept word, rare disease supplementary concept word, unique identifier, synonyms] |
| 42 | (Patient medical record* or PMR*).mp. [mp=title, abstract, original title, name of substance word, subject heading word, floating sub-heading word, keyword heading word, organism supplementary concept word, protocol supplementary concept word, rare disease supplementary concept word, unique identifier, synonyms] |
| 43 | (Personal health record* or PHR*).mp. [mp=title, abstract, original title, name of substance word, subject heading word, floating sub-heading word, keyword heading word, organism supplementary concept word, protocol supplementary concept word, rare disease supplementary concept word, unique identifier, synonyms] |
| 44 | (Personally controlled health management system* or PCHMS*).mp. [mp=title, abstract, original title, name of substance word, subject heading word, floating sub-heading word, keyword heading word, organism supplementary concept word, protocol supplementary concept word, rare disease supplementary concept word, unique identifier, synonyms] |
| 45 | (Problem-oriented medical record* or POMR*).mp. [mp=title, abstract, original title, name of substance word, subject heading word, floating sub-heading word, keyword heading word, organism supplementary concept word, protocol supplementary concept word, rare disease supplementary concept word, unique identifier, synonyms] |
| 46 | (Electronic health record* or EHR*).mp. [mp=title, abstract, original title, name of substance word, subject heading word, floating sub-heading word, keyword heading word, organism supplementary concept word, protocol supplementary concept word, rare disease supplementary concept word, unique identifier, synonyms] |
| 47 | (Source-oriented medical record* or SOMR*).mp. [mp=title, abstract, original title, name of substance word, subject heading word, floating sub-heading word, keyword heading word, organism supplementary concept word, protocol supplementary concept word, rare disease supplementary concept word, unique identifier, synonyms] |
| 48 | Time-oriented medical record*.mp. [mp=title, abstract, original title, name of substance word, subject heading word, floating sub-heading word, keyword heading word, organism supplementary concept word, protocol supplementary concept word, rare disease supplementary concept word, unique identifier, synonyms] |
| 49 | 32 or 33 or 34 or 35 or 36 or 37 or 38 or 39 or 40 or 41 or 42 or 43 or 44 or 45 or 46 or 47 or 48 |
| 50 | Open source.mp. [mp=title, abstract, original title, name of substance word, subject heading word, floating sub-heading word, keyword heading word, organism supplementary concept word, protocol supplementary concept word, rare disease supplementary concept word, unique identifier, synonyms] |
| 51 | (Adaptation* or implementation*).mp. [mp=title, abstract, original title, name of substance word, subject heading word, floating sub-heading word, keyword heading word, organism supplementary concept word, protocol supplementary concept word, rare disease supplementary concept word, unique identifier, synonyms] |
| 52 | Acceptance.mp. [mp=title, abstract, original title, name of substance word, subject heading word, floating sub- heading word, keyword heading word, organism supplementary concept word, protocol supplementary concept word, rare disease supplementary concept word, unique identifier, synonyms] |
| 53 | Adoption.mp. [mp=title, abstract, original title, name of substance word, subject heading word, floating sub- heading word, keyword heading word, organism supplementary concept word, protocol supplementary concept word, rare disease supplementary concept word, unique identifier, synonyms] |
| 54 | Alteration.mp. [mp=title, abstract, original title, name of substance word, subject heading word, floating sub- heading word, keyword heading word, organism supplementary concept word, protocol supplementary concept word, rare disease supplementary concept word, unique identifier, synonyms] |
| 55 | Fitting.mp. [mp=title, abstract, original title, name of substance word, subject heading word, floating sub- heading word, keyword heading word, organism supplementary concept word, protocol supplementary concept word, rare disease supplementary concept word, unique identifier, synonyms] |
| 56 | Integration.mp. [mp=title, abstract, original title, name of substance word, subject heading word, floating sub- heading word, keyword heading word, organism supplementary concept word, protocol supplementary concept word, rare disease supplementary concept word, unique identifier, synonyms] |
| 57 | Modification.mp. [mp=title, abstract, original title, name of substance word, subject heading word, floating sub- heading word, keyword heading word, organism supplementary concept word, protocol supplementary concept word, rare disease supplementary concept word, unique identifier, synonyms] |
| 58 | Usage.mp. [mp=title, abstract, original title, name of substance word, subject heading word, floating sub- heading word, keyword heading word, organism supplementary concept word, protocol supplementary concept word, rare disease supplementary concept word, unique identifier, synonyms] |
| 59 | 51 or 52 or 53 or 54 or 55 or 56 or 57 or 58 |
| 60 | 31 and 49 and 50 and 59 |
| 61 | 49 and 50 and 59 |
| 62 | 31 and 49 and 50 |
| 63 | 60 or 61 or 62 |
| 64 | limit 141 to (abstracts and english language and yr="1960 -Current") |

| **CINAHL** | |
| --- | --- |
| No. | **Searches** |
| S1 | (MH "Low and Middle Income Countries") OR (MH "Developing Countries") |
| S2 | (MH "World Health") OR (MH "Urban Health") OR (MH "Rural Health") OR (MH "Suburban Health") OR (MH "Social Determinants of Health") OR (MH "Population Health") OR (MH "Health and Disease") OR (MH "Public Health") |
| S3 | S1 OR S2 |
| S4 | TX LMIC* OR TX Constrained setting* OR TX Developing countr* OR TX Developing econom* OR TX Developing nation* OR TX Developing world OR TX Economically developing countr* OR TX Economically developing nation* OR TX Emerging countr* OR TX Emergent nation* OR TX Global south OR TX Informal settlement* |
| S5 | TX Limited resource setting* OR TX Low-resource setting* OR TX ( "Low and middle income countr*" ) OR TX Poor countr* OR TX Poor resource environment* OR TX Resource-constrained environment* OR TX Resource-limited setting* OR TX Resource poor setting* OR TX Resource poor environment* OR TX Slum setting* OR TX Slum population* OR TX Third World |
| S6 | TX Urban slum* OR TX Urban poverty OR TX Underserved environment* OR TX Underdeveloped nation* OR TX Underprivileged countr* |
| S7 | S3 OR S4 OR S5 OR S6 |
| S8 | (MH "Electronic Health Records+") OR (MH "Patient Record Systems+") OR (MH "Medical Records+") OR (MH "Medical Records, Personal") OR (MH "Health Information Systems+") OR (MH "Informatics") OR (MH "Clinical Information Systems") OR (MH "Problem Oriented Records") OR (MH "Nursing Records") OR (MH "Patient Discharge Summaries") OR (MH "Medical Record Linkage") OR (MH "Patient Portals") |
| S9 | TX ( electronic health record* or EHR* ) OR TX ( Computeri?ed patient record* or CPR ) OR TX ( County electronic health record* or CEHR* ) OR TX ( Electronic patient record* or EPR* ) OR TX ( Electronic medical record* or EMR* ) OR TX ( Electronic nursing record* or ENR* ) OR TX ( Medical record system* or MRS* ) OR TX ( National electronic health record* or National EHR* ) OR TX ( Nationwide electronic health record* or NEHR* ) OR TX ( Patient record system* or PRS* ) OR TX ( Patient medical record* or PMR*) ) OR TX ( Personal health record* or PHR* ) |
| S10 | TX ( Personally controlled health management system* orPCHMS*)ORTX( Problem-oriented medical record* or POMR* ) OR TX ( Source-oriented medical record* or SOMR* ) OR TX Time- oriented medical record* |
| S11 | S8 OR S9 OR S10 |
| S12 | TX open source |
| S13 | TX ( Adaptation* or implementation* ) OR TX Acceptance OR TX Adoption OR TX Alteration OR TX Fitting OR TX Integration OR TX Modification OR TX Usage |
| S14 | S7 AND S11 AND S12 AND S13 |
| S15 | S11 AND S12 AND S13 |
| S16 | S7 AND S11 AND S12 |
| S17 | S14 OR S15 OR S16 |
| S18 | S14 OR S15 OR S16 < Limiters - English Language; Abstract Available Search modes - Boolean/Phrase> |

| **Web of Science** | |
| --- | --- |
| No. | **Searches** |
| 1 | **TOPIC:** (LMIC* or Constrained setting* or Developing countr*) *OR* **TOPIC:** (Developing econom* or developing nation*) *OR* **TOPIC:** (Developing world or Economically developing countr*) *OR***TOPIC:** (Economically developing nation* or Emerging countr*) *OR* **TOPIC:** (Emergent nation* or Global south) *OR* **TOPIC:** (Informal settlement* or Limited resource setting*) *OR* **TOPIC:** (Low-  resource setting* or "Low and middle income countr*") *OR* **TOPIC:** (Poor countr* or Poor resource environment*) *OR* **TOPIC:** (Resource-constrained environment*or Resource-limited setting*) *OR* **TOPIC:** (Resource poor setting* or Resource poor environment*) *OR* **TOPIC:** (Slum setting* or Slum population*) *OR* **TOPIC:** (Third World or Urban slum*) *OR* **TOPIC:** (Urban poverty or Underserved environment*) *OR* **TOPIC:** (Underdeveloped nation* or Underprivileged countr*)  *Indexes=SCI-EXPANDED, SSCI, A&HCI, CPCI-S, CPCI-SSH, ESCI Timespan=1960-2021* |
| 2 | **TOPIC:** (Computeri?ed patient record* or CPR or County electronic health record* or CEHR*) *OR* **TOPIC:** (Electronic patient record* or EPR* or Electronic medical record* or EMR*) *OR* **TOPIC:** (Electronic nursing record* or ENR* or Medical record system* or MRS*) *OR* **TOPIC:** (National electronic health record* or National EHR* or Nationwide electronic health record* or NEHR*)  *OR* **TOPIC:** (Patient record system* or PRS* or Patient medical record* or PMR*) *OR* **TOPIC:** (Personal health record* or PHR* or Personally controlled health management system* or PCHMS*) *OR* **TOPIC:** (Problem-oriented medical record* or POMR* or Electronic health record* or EHR*) *OR* **TOPIC:** (Source-oriented medical record* or SOMR*) *OR* **TOPIC:** (Time-oriented medical record*)  *Indexes=SCI-EXPANDED, SSCI, A&HCI, CPCI-S, CPCI-SSH, ESCI Timespan=1960-2021* |
| 3 | TS=(open source)  *Indexes=SCI-EXPANDED, SSCI, A&HCI, CPCI-S, CPCI-SSH, ESCI Timespan=1960-2021* |
| 4 | **TOPIC:** (Adaptation* or implementation*) *OR* **TOPIC:** (Acceptance or Adoption or Alteration) *OR* **TOPIC:** (Fitting or Integration) *OR* **TOPIC:** (Modification or Usage)  *Indexes=SCI-EXPANDED, SSCI, A&HCI, CPCI-S, CPCI-SSH, ESCI Timespan=1960-2021* |
| 5 | #4 AND #3 AND #2 AND #1  *Indexes=SCI-EXPANDED, SSCI, A&HCI, CPCI-S, CPCI-SSH, ESCI Timespan=1960-2021* |
| 6 | #3 AND #2 AND #1  *Indexes=SCI-EXPANDED, SSCI, A&HCI, CPCI-S, CPCI-SSH, ESCI Timespan=1960-2021* |
| 7 | #4 AND #3 AND #2  *Indexes=SCI-EXPANDED, SSCI, A&HCI, CPCI-S, CPCI-SSH, ESCI Timespan=1960-2021* |
| 8 | #7 OR #6 OR #5  *Indexes=SCI-EXPANDED, SSCI, A&HCI, CPCI-S, CPCI-SSH, ESCI Timespan=1960-2021* |
