# Supplementary material for: Contextual Barriers to Implementing Open-Source Electronic Health Record Systems for Low- and Lower-Middle-Income Countries: Scoping Review
Source: J Med Internet Res. 2024 Aug 1;26:e45242. doi: 10.2196/45242 (PMC11327637; doi:10.2196/45242)
Supplement: Multimedia Appendix 3 [file jmir_v26i1e45242_app3.docx]

| World Bank List of LMICs [3] | | |
| --- | --- | --- |
| Economy | **Region** | **Income Group** |
| Algeria | Middle East & North Africa | Lower middle income |
| Afghanistan | South Asia | Low income |
| Angola | Sub-Saharan Africa | Lower middle income |
| Bangladesh | South Asia | Lower middle income |
| Benin | Sub-Saharan Africa | Lower middle income |
| Bhutan | South Asia | Lower middle income |
| Bolivia | Latin America & Caribbean | Lower middle income |
| Burkina Faso | Sub-Saharan Africa | Low income |
| Burundi | Sub-Saharan Africa | Low income |
| Cabo Verde | Sub-Saharan Africa | Lower middle income |
| Cambodia | East Asia & Pacific | Lower middle income |
| Cameroon | Sub-Saharan Africa | Lower middle income |
| Central African Republic | Sub-Saharan Africa | Low income |
| Chad | Sub-Saharan Africa | Low income |
| Comoros | Sub-Saharan Africa | Lower middle income |
| Congo, Dem. Rep. | Sub-Saharan Africa | Low income |
| Congo, Rep. | Sub-Saharan Africa | Lower middle income |
| Côte d'Ivoire | Sub-Saharan Africa | Lower middle income |
| Djibouti | Middle East & North Africa | Lower middle income |
| Egypt, Arab Rep. | Middle East & North Africa | Lower middle income |
| El Salvador | Latin America & Caribbean | Lower middle income |
| Eritrea | Sub-Saharan Africa | Low income |
| Eswatini | Sub-Saharan Africa | Lower middle income |
| Ethiopia | Sub-Saharan Africa | Low income |
| Gambia, The | Sub-Saharan Africa | Low income |
| Ghana | Sub-Saharan Africa | Lower middle income |
| Guinea | Sub-Saharan Africa | Low income |
| Guinea-Bissau | Sub-Saharan Africa | Low income |
| Haiti | Latin America & Caribbean | Lower middle income |
| Honduras | Latin America & Caribbean | Lower middle income |
| India | South Asia | Lower middle income |
| Indonesia | East Asia & Pacific | Lower middle income |
| Iran, Islamic Rep | Middle East & North Africa | Lower middle income |
| Kenya | Sub-Saharan Africa | Lower middle income |
| Kiribati | East Asia & Pacific | Lower middle income |
| Korea, Dem. People's Rep. East Asia & Pacific | | Low income |
| Kyrgyz Republic | Europe & Central Asia | Lower middle income |
| Lao PDR | East Asia & Pacific | Lower middle income |
| Lesotho | Sub-Saharan Africa | Lower middle income |
| Lebanon | Middle East & North Africa | Lower middle income |
| Liberia | Sub-Saharan Africa | Low income |
| Madagascar | Sub-Saharan Africa | Low income |
| Malawi | Sub-Saharan Africa | Low income |
| Mali | Sub-Saharan Africa | Low income |
| Mauritania | Sub-Saharan Africa | Lower middle income |
| Micronesia, Fed. Sts. | East Asia & Pacific | Lower middle income |
| Mongolia | East Asia & Pacific | Lower middle income |
| Morocco | Middle East & North Africa | Lower middle income |
| Mozambique | Sub-Saharan Africa | Low income |
| Myanmar | East Asia & Pacific | Lower middle income |
| Nepal | South Asia | Lower middle income |
| Nicaragua | Latin America & Caribbean | Lower middle income |
| Niger | Sub-Saharan Africa | Low income |
| Nigeria | Sub-Saharan Africa | Lower middle income |
| Pakistan | South Asia | Lower middle income |
| Papua New Guinea | East Asia & Pacific | Lower middle income |
| Philippines | East Asia & Pacific | Lower middle income |
| Rwanda | Sub-Saharan Africa | Low income |
| Samoa | East Asia & Pacific | Lower middle income |
| São Tomé and Principe | Sub-Saharan Africa | Lower middle income |
| Senegal | Sub-Saharan Africa | Lower middle income |
| Sierra Leone | Sub-Saharan Africa | Low income |
| Solomon Islands | East Asia & Pacific | Lower middle income |
| Somalia | Sub-Saharan Africa | Low income |
| South Sudan | Sub-Saharan Africa | Low income |
| Sri Lanka | South Asia | Lower middle income |
| Sudan | Sub-Saharan Africa | Low income |
| Syrian Arab Republic | Middle East & North Africa | Low income |
| Tajikistan | Europe & Central Asia | Lower middle income |
| Tanzania | Sub-Saharan Africa | Lower middle income |
| Timor-Leste | East Asia & Pacific | Lower middle income |
| Togo | Sub-Saharan Africa | Low income |
| Tunisia | Middle East & North Africa | Lower middle income |
| Uganda | Sub-Saharan Africa | Low income |
| Ukraine | Europe & Central Asia | Lower middle income |
| Uzbekistan | Europe & Central Asia | Lower middle income |
| Vanuatu | East Asia & Pacific | Lower middle income |
| Vietnam | East Asia & Pacific | Lower middle income |
| West Bank and Gaza | Middle East & North Africa | Lower middle income |
| Yemen, Rep. | Middle East & North Africa | Low income |
| Zambia | Sub-Saharan Africa | Low income |
| Zimbabwe | Sub-Saharan Africa | Lower middle income |

3. World Bank country and lending groups. World Bank. URL: [https://datahelpdesk.worldbank.org/knowledgebase/articles/906519-world-bank-country-and-lending-groups](https://eur03.safelinks.protection.outlook.com/?url=https%3A%2F%2Fdatahelpdesk.worldbank.org%2Fknowledgebase%2Farticles%2F906519-world-bank-country-and-lending-groups&data=05%7C02%7Cs.bostan%40leeds.ac.uk%7C595c373c5ec940ebaf8408dcab27f0f0%7Cbdeaeda8c81d45ce863e5232a535b7cb%7C0%7C0%7C638573435237266422%7CUnknown%7CTWFpbGZsb3d8eyJWIjoiMC4wLjAwMDAiLCJQIjoiV2luMzIiLCJBTiI6Ik1haWwiLCJXVCI6Mn0%3D%7C0%7C%7C%7C&sdata=Rvj1IPu6JS57bOseb%2FvViucVkHXQxRsMZNHRmqJb6eY%3D&reserved=0) [accessed 2021-09-01]
