# Supplementary material for: Contextual Barriers to Implementing Open-Source Electronic Health Record Systems for Low- and Lower-Middle-Income Countries: Scoping Review
Source: J Med Internet Res. 2024 Aug 1;26:e45242. doi: 10.2196/45242 (PMC11327637; doi:10.2196/45242)
Supplement: Multimedia Appendix 4 [file jmir_v26i1e45242_app4.docx]

**Inclusion and exclusion algorithm - for screening title and abstract, and full-text papers (level 1 and 2 screening):**

Yes, include article

Yes/maybe

Is the article about open source EHRs (all variations accepted)?

No 1

Reject article

Does the article include LMICs (all variations accepted)?

No 2

Reject article

No 4

Does the article report empirical data regarding the issues impacting the adaptation and /or non-empirical accounts of experiences and system descriptions in implementation of open source EHRs?

Reject article

Yes/maybe

Yes/maybe

Potentially relevant

Retrieve full text

Does the article cover any contextual barriers?

No 3

Reject article

Yes/maybe

No 5

Exclude article

Is the article relevant?
