# Supplementary material for: Contextual Barriers to Implementing Open-Source Electronic Health Record Systems for Low- and Lower-Middle-Income Countries: Scoping Review
Source: J Med Internet Res. 2024 Aug 1;26:e45242. doi: 10.2196/45242 (PMC11327637; doi:10.2196/45242)
Supplement: Multimedia Appendix 5 [file jmir_v26i1e45242_app5.docx]

| Characteristics of Included Studies | | | | | | | |
| --- | --- | --- | --- | --- | --- | --- | --- |
| Author(s)/ Publication year | **Country of Origin** (where the study was conducted) | **Aim(s)/ Purpose** | **Study Design** | **Study Population/ Sample Size** | **Methods** | **Intervention Type (Open-source EHR)** | **Key Findings** |
| Mohammed-Rajput et al. (2011) [9] | Kenya, Rwanda, Lesotho, Tanzania, Uganda, and Malawi | To examine experiences of OpenMRS implementers who work in resource-constrained settings; addressing the barriers and facilitators to implementation. | Qualitative  Study | Purposive sampling used; 10 sites across 7 countries in Africa, sites ranging from small (450 patients) to large (95,000 patients). Including clinical and non-clinical staff. Mainly resource-constrained (rural) settings. | Multimethod approach: qualitative and quantitative. Used interviews (in-person and telephone), focus groups and an electronic survey. | OpenMRS | Key challenges impacting implementation: power, human resources (staffing and training), incomplete data, funding, software limitations and documentation. |
| Syzdykova et al. (2017) [24] | Ethiopia | To conduct a systematic review of open-source EHR systems in low resource settings. | Systematic Review | Evaluation and comparison of five open-source EHR systems for low-resource (rural) settings. | Evaluation methodology using two predefined criteria’s:  1) 21 defined system features (user requirements) formed through discussions with physicians and research network partners and publication analysis.  2) five OSEHR systems selected through a literature search using three databases with defined key terms.  *Five systems were installed and evaluated against the defined requirements. | GNU Health, OpenEMR, FreeMED, OpenMRS, and Bahmni | Various contextual barriers addressed: lack of offline support (loss of filled-in data), potential concerns for security and privacy with adaptation, technical expertise (programing) and advance computer skills required, lack of guides and documentation. |
| Muinga et al. (2018) [21] | Kenya | To present a descriptive case study of an open-source EHR implementation in Kenya. | Qualitative  Study | Covered public health care facilities such as hospitals in rural Kenya; site visits to county hospitals in Machakos. | A landscape review of existing literature (eHealth policies and EHR development in Kenya). Five site visits to county hospitals: conducting semi-structured and informal interviews, attending project meetings and group discussions with consultants, implementers and MoH.  Implemented an open-source EHR system across counties in Kenya (two versions). | OpenMRS: KenyaEMR, AfeyaEHMS | Key challenges impacting implementation: lack of power, data security functionality, sustainability issues, limited interoperability or integration with other systems, limited technical expertise, lack of system support and user buy-in, human resources (staffing), no leadership, lack of stakeholder engagement and lack of system ownership.  It is practical to implement on a focused scale rather than implementing on broad scale – which can be a major challenge |
| Oza et al. (2017) [28] | Sierra Leone | To describe work conducted on a rapid developing open-source Ebola EHR system in Sierra Leone. | Quantitative  Study | Implementation of OpenMRS-Ebola in Kerry Town ETC, rural district of Sierra Leone from November 2014 – March 2015. An international mix of 100 clinicians commissioned. | Used Agile software methodology: recruitment of individuals with diverse skills and experiences, iterative design, phased implementation (phases 1, 2, and 3), effective communication and feedback. For system evaluation questionnaires were used. | OpenMRS-Ebola v1.10.3 | Phase 1 was successful, however phases 2 and 3 were not implemented as the no. of new Ebola cases decreased leading to closures of the ETC (uncontrollable external factors) limiting usefulness of the system.  Key challenges: system design (UI), power issues, inadequate training, technical skills, funding, technical delays (teething issues), duplication of work, incomplete drug list, integration issues (parallel use of paper and EHR systems), communication difficulties between zones, time issues and staffing challenges. |
| Akanbi et al. (2012) [44] | Sub-Saharan Africa | To review the availability of EHRs in sub-Saharan Africa and highlight challenges inhibiting implementation. | Systematic Review | 21 articles out of 147 were reviewed. Most from sub-Saharan Africa. | Two databases were searched: PubMed and Google Scholar using five terms, with the inclusion criteria of ‘use of EHR in Africa’. | OpenMRS | The key challenges with implementation were: high cost of set-up and maintenance, poor network and power infrastructure for remote locations (rural), resistance and lack of confidence from staff, low morale, workload issues, over-reliance on funders (international partners), scarce government support.  91% of the articles reported wide use of OpenMRS. |
| Fish and Guha (2020) [68] | Haiti | To review requirements for development of an open source electronic medical record system for a rural Haitian medical clinic. | Descriptive study (accounts of experiences/system descriptions) | Plan to implement an open-source EHR system (OpenMRS) in a rural Haitian medical clinic.  Currently, OpenMRS is implemented in eight clinics/ hospitals in Haiti. | Descriptive analysis of proprietary versus open source software. Reviewing examples and projects that use open source EHRs in LMICs – identify implementation challenges and opportunities to develop own implementation project. Plan to pilot test an open source EHR system for a rural Haitian medical clinic, using a single computer – with a foreign NGO providing funding for the project. Recommend using open source software/ OpenMRS for rural primary clinics in Haiti. | OpenMRS | The key challenges observed were: sustainability issues, poor internet connectivity, lack of computer training, lack of facility management support, financial concerns (funding), poor system design not fir for environment, no involvement of end-users, and poor system security.  Emphasise the importance of addressing cultural and health related issues - a key priority: instilling a culture whereby clinicians’ consider patient history, record patient data/ capture information in real time. |
| Verma et al. (2021) [83] | Kenya, Nepal, Liberia, Lesotho, Haiti, Uganda, Sierra Leone, Rwanda, Nigeria, Mozambique, Malawi, Kazakhstan, India, Ethiopia, Democratic People’s Republic of Korea, and Peru | To understand the impact that OpenMRS as a Global Good has created. It also aimed to understand key challenges, and unmet needs to understand where continued investment is needed. | Mixed Methods Study | OpenMRS Atlas has data from 381 sites over a reporting period from 11/11/2012 to 12/15/2019 from 87 countries. Mainly resource-constrained (rural, remote) settings.  A total of 26 organizations from 62 countries submitted data about their implementations to the annual OpenMRS report.  Sixteen implementations across 10 organizations responded to the survey. | Three data sources utilised: OpenMRS Atlas, community annual report, and implementer survey (includes both quantitative and qualitative questions). | OpenMRS | Key challenges with implementation: finding technically skilled staff, resistance to change – acceptance of EHRs, lacking in availability of key resources (internet/ power, computers), inadequate training, poor change management – transition from paper to electronic use, lack of support and poor documentation – inconsistencies. |
| Jawhari et al. (2016) [84] | Kenya | To explore the perceptions of stakeholders exposed to an open-source EMR in two urban slum clinics; addressing the barriers and facilitators. | Qualitative  Study | Two urban-poor slum primary care clinics in Kibera, Nairobi, newly adopting an open-source EMR. The sample was purposeful focusing on: nurses, physicians and support staff. | Descriptive qualitative method used: open-ended and semi-structured interviews. 10 participants asked to share their observations on use of the EMR. | Open-source EMR (OpenMRS) | There are diverse challenges associated with adaptation and implementation of open-source EMR in slum settings. Key challenges grouped in three themes: systems (power, infrastructure, interoperability), software (confidentiality and security), social (identity management and change management). |
| Gainer et al. (2012) [100] | Ethiopia | To present a standards-based open-source EHR software application, designed for use in resource constrained environments. | Descriptive study (accounts of experiences/system descriptions) | A pilot tested conducted in 2011 by HR4E, on school children in Yetebon, a rural community in Ethiopia. | Health assessments were undertaken over six days for over 1100 children in mobile clinics. A team of 21 members supporting the implementation across stations (using five configured laptops): physicians, nurses, nurse-translators and IT support from the US and Ethiopia. Patients visited the clinic with a paper record and a USB-drive (attached to the wrist) containing an electronic form of the record. | HR4E application (open-source - Django) | Key challenges were: skills, power, connectivity and extensibility. The lack of robustness of the software; not supporting efficient annotations. Certain laptops were reported as major inhibitors; users lacking technical skills and confidence, lack of IT support. Workflow in busy environments can impede adaptation and implementation. |
| Gyamfi et al. (2017) [101] | Ghana | To identify the facilitators and barriers to implementing EMR in an emergency center. | Qualitative  Study | 24 participants: implementers and end-users at KATH hospital in Ghana (urban area). | A descriptive, cross sectional design using semi-structured interviews (in English). Data analyse using thematic analysis. | OpenEMR v4.1.0 | The key barriers were: financial resources, data security, logistical constraints, human resources, motivation, data backup and power instability. The lack of funding significantly limit EMR adaptations and implementations. |
| Were et al. (2010) [102] | Uganda | To describe an alternative accessible model for implementing EHRs in resource limited settings. | Quantitative  Study | Three OpenMRS implementation sites (Masaka, Mbale and Mbarara) in Uganda (resource-poor urban settings). | Conducted time-motion studies in Masaka to assess impact of the EHR pre- and post-implementation. Surveys (point scale) were used to evaluate user attitudes towards the EHR implementation using the proposed model (TEC): convenience sample of 45 clinical providers. Controlled observations were conducted with clinicians and patients pre- and post-implementation. | OpenMRS | A few users reported dissatisfaction with the support infrastructure, need for further training and an increase in waiting time post-implementation; EHRs perceived to be slower than paper method. Time-motion studies were performed at one site only, could potentially limit generalizability of findings.  Sites that are considered too small and resource constrained are not able to support autonomous implementations; restricting self-sufficiency. |
| Anantraman et al. (2002) [103] | India | To describe the technical challenges and innovation required in the design, development, adaptation and implementation of a handled EMR in rural India. | Descriptive study (accounts of experiences/system descriptions) | Implemented in Northern India: 28,000 people living in 11 rural villages – covered by four paramedics. | Paramedics were selected based on experience: early and late adopters.  Training the paramedics in two phases to understand and use the system (handheld EMR). Training provided by two students and three doctors locally. | Open-source EMR (Linux operating system) | Implementation is best achieved by involving end-users in the design (key stakeholders). There are potential costs issues which should be assessed after the system has been in use over time. The major limitation is data security. Issues with pre-implementation, go-live and post-implementation in rural areas.  The training was offered in the local language which helped the paramedics understand and use the system – context specific. |
| Raut et al. (2017) [104] | Nepal | To describe the design and implementation of an EMR at a public hospital in rural Nepal. | Descriptive study (accounts of experiences/system descriptions) | 30 participants at Bayalpata hospital – three groups: five doctors, 13 mid-level providers and 12 nurses. | Conducted focus group discussions with each group. Used anonymous surveys (questions scored on a scale). | Bahmni: OpenERP, OpenMRS, OpenELIS | Key challenges with implementation: technology inhibiting patient-provider relationship, power-cuts causing extra work (reverting to paper use), confusion with responsibilities (clinician role in documenting), human resource (staff training), infrastructure issues and lack of trust with the system. |
